# Effective Delivery of Arsenic Trioxide to HPV-Positive Cervical Cancer Cells Using Optimised Liposomes: A Size and Charge Study

**DOI:** 10.3390/ijms19041081

**Published:** 2018-04-04

**Authors:** Anam Akhtar, Scarlet Xiaoyan Wang, Lucy Ghali, Celia Bell, Xuesong Wen

**Affiliations:** Department of Natural Sciences, Middlesex University, The Burroughs, Hendon, London NW4 4BT, UK; a.akhtar@mdx.ac.uk (A.A.); x.wang@mdx.ac.uk (S.X.W.); l.ghali@mdx.ac.uk (L.G.); c.bell@mdx.ac.uk (C.B.)

**Keywords:** arsenic trioxide (ATO), liposome, drug delivery, cervical cancer, human papilloma virus (HPV)

## Abstract

Despite the success of arsenic trioxide (ATO) in treating haematological malignancies, its potential to treat solid tumours has not been fully exploited, owing to its dose-limiting toxicity and poor pharmacokinetics. In order to overcome this hurdle, liposomal encapsulation of the drug with different surface charges (neutral, negative, and positive) and sizes (100, 200 and 400 nm) were synthesised and tested on human papilloma virus (HPV)-positive HeLa and HPV-negative HT-3 cervical cancer cell lines. Two epithelial cell lines—human keratinocytes (HK) and human colon cells (CRL-1790)—were used as controls. The synthesised liposomes were tested for their physico-chemical characteristics, drug loading efficiency, and toxicity on the studied cell lines. Neutral liposomes of 100 nm in size were the chosen formulation for delivering ATO into the studied cells, as they showed the least intrinsic cytotoxicity and the highest loading efficiency. The findings demonstrated that the optimised formulation of liposomes was an effective drug delivery method for HPV-infected cervical cancer cells. Furthermore, the toxicity vs. uptake ratio was highest for HeLa cells, while a reduced or minimal toxic effect was observed for non-HPV-infected cervical cancer cells and control cells. These findings may provide a promising therapeutic strategy for effectively managing cervical cancers.

## 1. Introduction

Cervical cancer is responsible for 14% of all gynaecological cancers in women, and is linked to high-risk human papilloma virus (HPV) infections [1,2,3]. Among all of the high-risk HPV subtypes, HPV-16 and HPV-18 account for 70% of all cervical cancers [4,5]. Invasive cervical cancer management depends on the stage of the cancer, histological type, and the age of the patient. The most common treatment is surgery and/or chemoradiotherapy, which usually results in severe side effects [6,7]. Therefore, alternative treatment options that provide better efficacy and specificity with fewer side effects on the surrounding healthy tissues and cells are necessary. An anti-HPV agent that can be taken up more specifically by HPV-infected cells is needed because of the crucial role of high-risk HPV infections in the pathogenesis of cervical cancer. In addition, if a drug carrier can deliver the drug to the cancer cells and release it slowly, a consistent flow of medication—which may enhance the therapeutic effect of the delivered drug—could be maintained. Moreover, the toxic effects on non-HPV-infected surrounding cells and tissues would be reduced. 

Arsenic trioxide (ATO) was approved by the Food and Drug Administration (FDA) in 2000 as a first-line treatment option for acute promyelocytic leukaemia (APL). It has also been found to be effective for other haematological malignancies in vitro [8,9,10,11]. Although ATO has displayed some anti-cancer effects in vitro, poor pharmacokinetics and its dose-limiting toxicity have caused it to not exhibit appreciable clinical success on human patients when its efficacy was tested for solid tumours [11]. In order to overcome this difficulty and improve ATO’s anti-cancer properties for solid tumours (specifically cervical cancer), a mechanism is needed for increasing the drug’s therapeutic index by sparing the surrounding healthy tissue from arsenic toxicity. This could be achieved by encapsulating the drug using nanotechnology. 

Nanotechnology-based drug delivery systems can specifically target cancer cells while avoiding their healthy neighbours and avoiding rapid clearance from the body, thereby considerably increasing the therapeutic index of the drug [6,12]. Among the many nanocarriers that have been discovered to date, the oldest and most commonly employed nanoparticles are liposomes [13,14,15]. Chen et al. (2006) utilised the “remote loading phenomenon” to stably encapsulate ATO in liposomes. As arsenic tends to combine with transition metal ions such as Ni^II^, Co^II^, and Pt^II^ to form a precipitate, liposomes were loaded with an acetate solution of Ni prior to the addition of ATO [16]. An efflux of protonated acetic acid drove ATO to be efficiently loaded and stably encapsulated within the liposomes, with the transition metal ion in the form of a solid crystalline precipitate [16].

Although they are an exciting and promising prospect, optimisation is required in order to use liposomes as a desirable drug delivery vehicle. There are many physicochemical characteristics that need to be considered, including size, surface charge, zeta potential, shape, membrane lipid packing, steric stabilization, and polyethylene glycol (PEG) fluidity. These features influence the pharmacokinetic properties of liposomes by controlling the clearance process, surface toxicity, mononuclear phagocytic system (MPS) recognition, and enhanced permeability and retention effect (EPR) [17,18]. Among these factors, size and surface charge are the two key characteristics of nanoparticles that affect their EPR, immune clearance, and cellular adhesion and uptake [19].

While a few studies have investigated the effects of both the surface charge and size of liposomes on cellular uptake, the results have been inconsistent [19,20,21,22,23]. Regarding surface charge, Nie et al. [19] reported that positively-charged cationic liposomes were more readily taken up by cells—both in vitro and in vivo—because of the interaction with the negatively-charged cell membrane. In contrast, Miller et al. [20] found that a negative surface charge on liposomes increased endocytosis by some cell types. An earlier study reported that liposome–cellular interaction depends not only on the liposomal charge, but also on the lipid composition and—more importantly—the cell type [21]. Therefore, it is crucial to optimise the liposomal construct in order to identify the best charge for the target cancer cell line under investigation. 

An optimum size for nanoparticles is also important, as the kidneys rapidly clear any nanoparticle with a size below 10 nm, while those above 150 nm in size would face the risk of recognition and clearance by the body’s immune system [22]. However, larger nanoparticles have been reported to benefit from having higher encapsulation efficiencies, which increases drug delivery to the target tissue [23]. 

Based on the inconsistency of previous findings, the aim of this study was to identify the most suitable liposomal formulation—with respect to size and charge—for delivering ATO into cervical cancer cells, and thereby enhance the therapeutic potential of the delivered drug. The uptake and anti-tumoural efficacy of ATO using the optimised liposomal formulation were examined using two cervical cancer cell lines—namely HeLa (HPV-positive) and HT-3 (HPV-negative)—along with two cell lines as controls—namely human epidermal keratinocytes (HK) and human colon epithelial cells (CRL-1790). 

## 2. Results

### 2.1. Liposome Preparation and Characterization

The mean size and zeta potential of control liposomes were determined by dynamic light scattering (DLS) on a Zetasizer-Nano ZS (Malvern Instruments, Malvern, UK). The DLS measurements of the hydrodynamic sizes of the 100 nm, 200 nm, and 400 nm liposomes were 138.5 ± 1.22 nm, 187 ± 2.96 nm, and 243 ± 2.68 nm, respectively. All of the charged liposomes were extruded through a 100 nm filter. Positively-charged and neutral phosphatidylcholine (PC) liposomes had a similar hydrodynamic size of 138.5 ± 1.22 nm and 143.9 ± 3.19 nm, respectively. However, the incorporation of a negatively-charged lipid (DSPG: 1,2-distearoyl-sn-glycero-3-phospho-(1′-rac-glycerol), sodium salt) slightly increased the particle size to 165.2 ± 3.26 nm. No significant differences were observed between these three sizes. The polydispersity index of the investigated vesicles had values ranging from 0.12 to 0.175, indicating a homogenous population of liposomes. Regarding the zeta potential, the group of liposomes that exhibited a small negative surface charge, (−) 24.4 ± 0.46 mV was considered neutral. Cationic liposomes possessed a positive charge, (+) 23.2 ± 0.44 mV, and negative liposomes possessed a higher negative charge, (−) 36.4 ± 0.75 mV.

**Figure 1:**
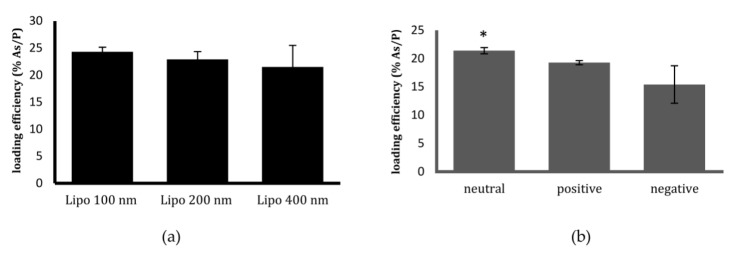
Loading efficiency of synthesised liposomes by inductively coupled plasma optical emission spectrometry (ICP-OES). Loading efficiency (% phospholipids/encapsulated arsenic—As/P) of liposomes of (**a**) different sizes and (**b**) different charges. All of the different-sized liposomes (100 nm, 200 nm, and 400 nm) have a neutral charge, whereas all of the different-charged liposomes (neutral, positive, and negative) are 100 nm in size. Data are presented as mean ± standard deviations (SD) of three replicate measurements of at least three independent experiments. An unpaired *t* test (*p* > 0.05) was used to test for any significant difference in the loading efficiency of liposomes of three sizes (ranging from 100 to 400 nm), and three charges (neutral, negative, and positive). No significant difference was observed between the liposomes of different sizes, although neutral liposomes displayed a significantly higher loading efficiency than the others (* *p* < 0.05).

The liposomes were efficiently loaded with arsenic, which was co-encapsulated with the transition metal ion, Ni. The phospholipids—encapsulated arsenic and nickel—in the liposomes were quantified by inductively coupled plasma optical emission spectrometry (ICP-OES). The loading efficiencies (phospholipids/encapsulated arsenic—As/P) of liposomes of different sizes and charges are presented in Figure 1. The loading efficiency was highest for neutral liposomes of 100 nm in size at 24.2% As/P (±0.848).

**Figure 2:**
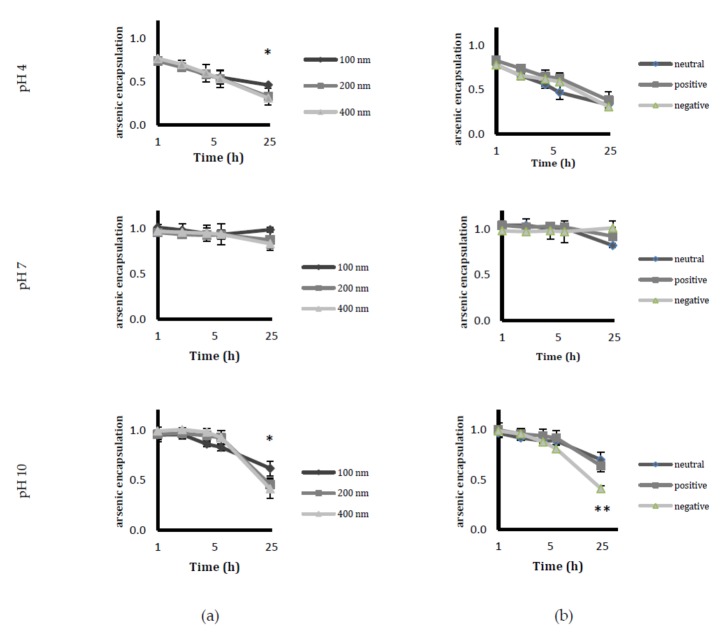
Stability studies of different liposomal formulations under various pH conditions. Arsenic trioxide (ATO) was encapsulated in liposomes of (**a**) different sizes and (**b**) different charges after dialysing in buffers at pH 4, pH 7, and pH 10. Data are shown as mean ± SD of three independent experiments; * *p* < 0.05, ** *p* < 0.01.

In order to assess the effect of pH on the liposomal formulation (and hence determine the drug leakage pattern that is initiated when encountering different pH), liposomes were dialysed in buffers of pH 4, pH 7 and pH 10. The amounts of the drug that were retained in the liposomes were examined after periods of 1, 2, 4, 6, and 24 h (Figure 2). At pH 4, approximately 40% of the drug was lost within the first four hours. Among the different sizes, the smallest (100 nm) liposomes were found to be the most stable at all pH values. With respect to charge, the negatively-charged liposomes displayed a significant loss of stability when they were exposed to a higher pH in comparison with those with a neutral or positive charge. 

### 2.2. Analysing Cytotoxicity of Control Empty Liposomes with Different Sizes and Charges

**Figure 3:**
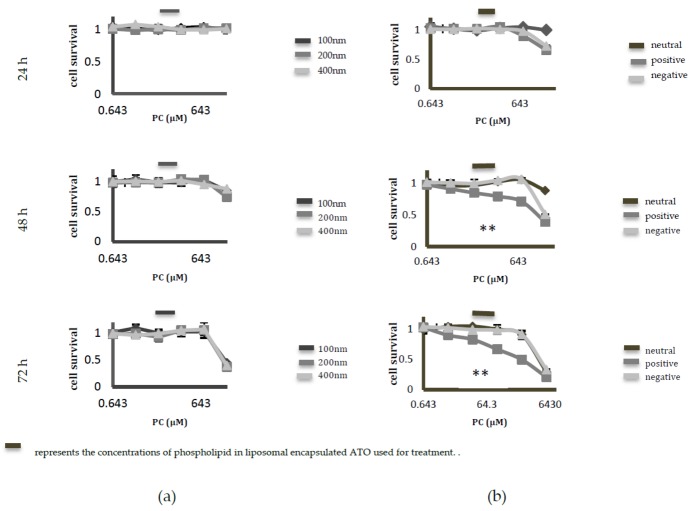
The MTT assay used to test the cytotoxicity of various control liposomal formulations on cervical cancer cells. The cellular toxicity that is induced by control (empty) liposomes of different (**a**) sizes and (**b**) charges is represented following an incubation period of 24, 48 and 72 h with HeLa cells. The positively-charged liposomes displayed noticeable toxicity at 48 h exposure and at the same dilution factor that was used for diluting liposomal encapsulated ATO. Neutral liposomes were found to show the least toxicity. Data are presented as mean ± SD of three replicate experiments; ** *p* < 0.01. PC: phosphatidylcholine.

Control empty liposomes of various formulations were synthesised and tested—using the 1-(4,5-Dimethylthiazol-2-yl)-3,5-diphenylformazan (MTT) assay—for their cytotoxicity towards HeLa cells at 24, 48 and 72 h (Figure 3). The phospholipid concentrations of the liposomes were diluted at the same dilution factor that was used for liposomal ATO. No significant difference in the cytotoxicity from different-sized liposomes was observed at the relevant concentrations of liposomes. However, when the surface charges were taken into consideration, the empty positively-charged liposomes displayed significant toxicity over an incubation period of 48 h.

### 2.3. Cytotoxicity and Uptake of ATO-Encapsulated Liposomes in HPV-Positive and HPV-Negative Cervical Cancer Cell Lines

After establishing that neutral liposomes of 100 nm in size were the most stable formulation, possessed the highest encapsulation efficiency, and displayed the least intrinsic toxicity, this form of liposome was chosen as the drug carrier for the remainder of the experiments. The response of cervical cancer cell lines of differing HPV statuses (HPV-positive HeLa and HPV-negative HT-3) to the treatment with ATO—delivered either in the free form or encapsulated in the chosen liposomes—was investigated with regards to cytotoxicity (MTT assay), cellular uptake (inductively coupled plasma mass spectrometry, ICP-MS), and induction of apoptotic response (flow cytometry). 

**Figure 4:**
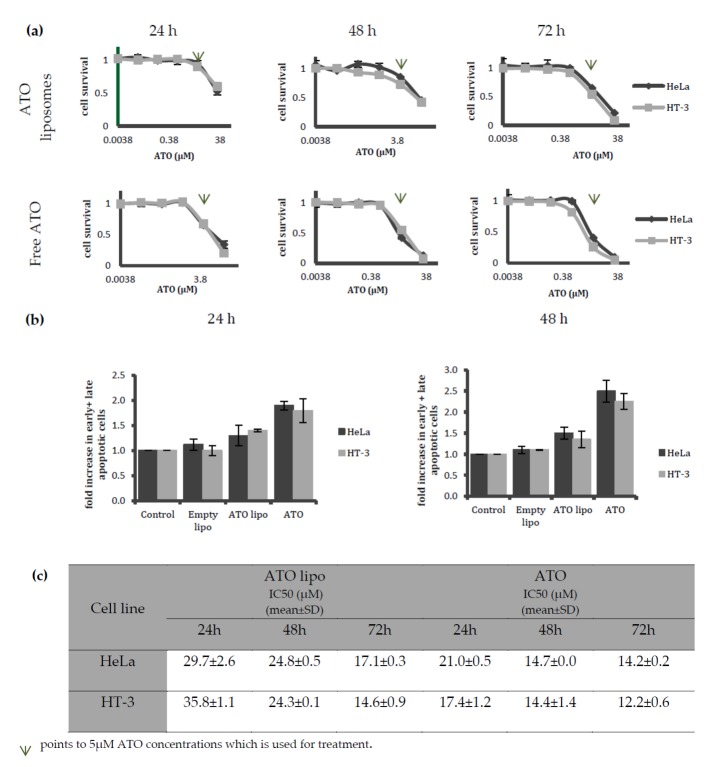
The MTT assay and flow cytometry analysis to compare cellular toxicity and apoptosis induction of varying treatments on HeLa and HT-3 cells. (**a**) Cell viability of HeLa and HT-3 cell lines following incubation with liposomal encapsulated ATO (ATO lipo) and free ATO for 24, 48 and 72 h; (**b**) Flow cytometry analysis of apoptotic populations that were treated with complete media as control, empty liposomes, liposomal 5 µM ATO, or 5 µM free ATO on HeLa and HT-3 cell lines following treatment for 24 and 48 h; (**c**) Calculated IC_50_ values for ATO lipo and free ATO treated HeLa and HT-3 cells after 24, 48, and 72 h. Data are presented as mean ± SD of three replicate experiments.

The MTT results demonstrated that the cell survival rates after treatment in both cervical cancer cell lines were similar for up to 72 h (Figure 4a). In addition, the cell survival rates were found to be lower in cells that were exposed to free ATO as opposed to liposomal-encapsulated ATO. This trend became more noticeable as the drug exposure time increased. Using flow cytometry to measure apoptosis (Figure 4b), no statistically significant difference was found between the apoptotic populations in HT-3 and HeLa cell lines after incubation with either free or encapsulated ATO for up to 48 h treatment (Figure 4b). However, a clear trend of increased cell death induced by directly exposing both of the cell lines to free ATO was observed. The IC_50_ results that were obtained from the cytotoxicity assays are presented in Figure 4c. The results display similar values for both of the treatments on HeLa and HT-3 cells after 48 h of incubation.

**Figure 5:**
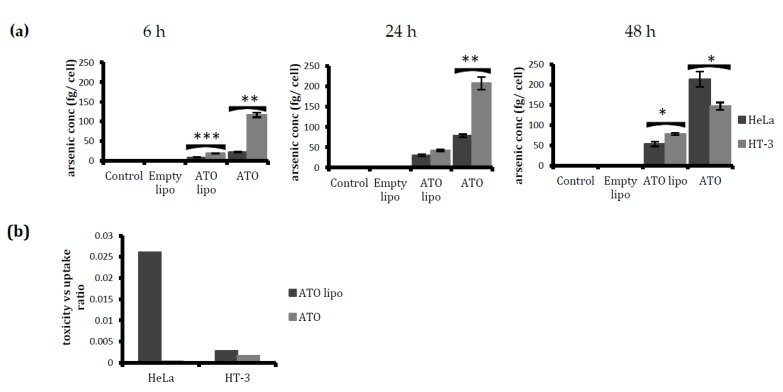
A comparative study of the arsenic uptake and toxic response of different drug formulations on HeLa and HT-3 cells. (**a**) Comparison of the arsenic uptake by HeLa and HT-3 after 6, 24, and 48 h of treatment—as determined by inductively coupled plasma mass spectrometry (ICP-MS). Free ATO was taken up more than ATO lipo in both cell lines. However, the uptake was higher in HT-3 for all treatments, except for the free ATO uptake after 48 h. (**b**) Ratios of toxicity vs. uptake of liposomal and free ATO for the two cervical cancer cell lines after 24 h treatment. Data are displayed as mean ± SD from at least three independent experiments; * *p* < 0.05, ** *p* < 0.01, and *** *p* < 0.001.

Despite a similar response to treatment with free and encapsulated ATO from both of the cell lines with respect to toxicity and induction of apoptosis, the uptake of both free and liposomal arsenic by the cell lines varied at different time points (as can be observed in Figure 5a). The uptake by HT-3 cells was found to be higher than the uptake by HeLa cells at 6 and 24 h. This trend reversed for the free drug uptake at 48 h treatment, when HeLa cells were observed to have taken up significantly more ATO. In Figure 5b, toxicity–uptake ratios for HeLa and HT-3 are shown to indicate the level of toxicity that was induced in the cell lines per unit of arsenic taken up by the cells. Liposomal ATO was observed to be more effective than the free drug in inducing toxicity per unit of arsenic uptake in both cell lines. However, liposomal ATO treatment was more toxic to HeLa cells than to HT-3 cells. 

### 2.4. Selectivity of Liposomal Encapsulated ATO in Killing Cervical Cancer Cells

**Figure 6:**
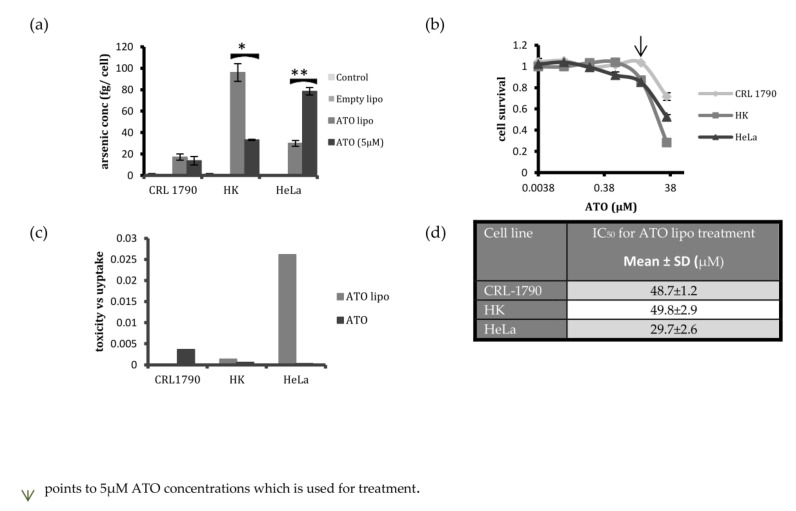
Arsenic uptake and toxic response in the control cells and HeLa cells. (**a**) A comparative study of arsenic concentration per cell in CRL-1790, human epidermal keratinocyte (HK), and HeLa—as determined via ICP-MS after 24 h of treatment with media, control liposomes, ATO lipo, and free ATO; (**b**) Cell survival following the exposure of cervical cancer and control cell lines to ATO lipo for 24 h—assessed using the MTT assay; (**c**) Toxicity–uptake ratio of ATO lipo and free ATO in HeLa cells compared to control cell lines, after treatment for 24 h. ATO lipo is effective in generating toxicity in HeLa cells, while having a non-toxic effect on the normal cells; (**d**) The IC_50_ values for three cell lines following 24 h of ATO liposomal treatment. Data are displayed as mean ± SD from three replicates; * *p* < 0.05, ** *p* < 0.01.

After establishing the efficacy of liposomal ATO in inducing toxicity per unit of arsenic uptake in HPV-positive HeLa cells, we further investigated the effect of liposomal ATO on non-cancerous cells by choosing two cell lines as controls, namely human keratinocytes (HK) and human colon cells (CRL-1790) (Figure 6a,b). Among the three cell lines investigated, the uptake of liposomal arsenic was highest in HK cells, with a lower uptake by HeLa and CRL-1790 cells. In contrast, the highest uptake of free ATO was by HeLa cells (Figure 6a). However, when cytotoxicity was investigated, cell survival was insignificantly affected in CRL-1790 and HK cell lines (Figure 6b). The ratio of toxicity to the amount of arsenic taken up by the cells after incubation with liposomal encapsulated ATO and free ATO for 24 h is presented in Figure 6c. Liposomal ATO resulted in a higher cell death rate per unit uptake of arsenic than free ATO for HeLa cells, while a minimal toxic effect was observed in the control cells. The IC_50_ values for the liposomal ATO treatment were calculated, and were higher for the control cell lines than for HeLa cell lines (Figure 6d).

## 3. Discussion

Since the recognition of the potential of ATO as a broad spectrum anti-cancer drug, it is being increasingly explored for the treatment of various cancer types [11]. However, the systemic toxicities that are associated with this drug when treating solid cancers impedes its therapeutic use in clinical applications. We have previously shown that a low concentration of ATO (≤2 µM) is capable of selectively inducing cellular apoptosis and increasing p53 expression in HPV-positive cervical cancer cells in vitro [24]. However, increasing the ATO dosage to 5 µM resulted in most of the cells being killed because of drug-induced toxicity, irrespective of their HPV status. In order to reduce ATO’s toxicity and raise its therapeutic index, liposomal encapsulated ATO at 5 µM was used to treat cervical cancer cells [25]. The results from our previous study indicated that, although liposomal ATO was less readily taken up by cells than free ATO [26], the delivered ATO was able to reduce the oncogene E6 expression while exhibiting reduced toxicity [25]. Since the size and surface charge of liposomes play important roles in drug stability and sustained release, an optimised liposome formulation with appropriate physical features would be essential for further improving the drug loading efficiency and boosting the killing effects for cancer cells. Therefore, the aim of this work was to optimise the liposomal design—with respect to size and charge—for use as drug delivery vehicles to specifically target HPV-positive cervical cancer cells in vitro. Liposomes of different sizes (100 nm, 200 nm, and 400 nm) and different charges (neutral, positive, and negative) were synthesised, with ATO co-encapsulated with a transition metal ion (Ni^2+^) in a stable, precipitate form.

The size measurement analysis indicated a disparity between the actual and expected sizes of the formed liposomes. The 200 nm liposomes were closest to the expected size, with the 100 nm liposomes being slightly larger and the 400 nm liposomes being smaller. This could be caused by the choice of filter extruders (discontinuous syringe filtration vs. continuous high-pressure devices), the inaccuracy of the actual pore diameter of the commercial filter, or the particle size analysis technique that was employed [27]. However, as these liposomes still displayed an expected incremental size range, it was decided to proceed with the planned analysis. 

Circulating liposomes with stability in the retention of drugs is a desirable feature in order to ensure that a sufficient amount of the drug reaches the target tissue. The pH stability studies were carried out under three pH conditions (pH 4, pH 7 and pH 10). Among the studied sizes, 100 nm liposomes were the most stable at all three pH values. Negative liposomes displayed instability at higher pH, most likely owing to the changes in lipid orientation in the alkaline conditions leading to liposome disintegration [28]. 

The results from the drug loading efficiency studies among different formulations indicated that 100 nm neutral liposomes had the highest drug encapsulation. Since the success of nanoparticles as drug carriers relies on them being inherently non-toxic, we further tested whether these liposomes displayed any intrinsic toxicity towards the cells. This was tested by incubating their empty counterparts with HeLa cells for 24, 48 and 72 h. The dilutions of liposomes that were tested for toxicity studies were the same as the dilutions that were used for preparing liposomal encapsulated ATO samples. Size did not have any significant effect on toxicity in the range that was tested (Figure 3a). However, among the charged liposomes, positively-charged liposomes began to display toxicity to HeLa cells after 48 h of incubation, killing almost 20% of cells at the dilutions that were employed for the treatment (Figure 3b). One possible reason suggested for the high toxicity of cationic lipids is their translocation to the cell membrane, which eventually results in its destabilisation [29]. This finding excludes positively-charged liposomes from being employed as drug carriers for ATO delivery to cervical cancer cells, even though a higher uptake by cells was previously observed with this type of liposome [19]. Moreover, negatively-charged liposomes were demonstrated to be unstable at a higher pH. Considering the above reasons, 100 nm, neutral liposomes were chosen as the carrier for ATO delivery and were used for further in vitro analysis.

Further investigations on cytotoxicity and apoptosis induced by treatments with liposomal ATO and the free drug indicated a trend of toxicity increasing with drug exposure time in both of the cervical cancer cell lines that were tested (Figure 4a,b). An ICP-MS examination of the amount of ATO that was taken up by cells indicated that, using both delivery methods, HT-3 cells took up more arsenic than HeLa cells (Figure 5a). The ratio of toxicity to uptake indicates that liposomal ATO was 71 times more toxic to HeLa cells than to HT-3 cells per unit of arsenic. In contrast, free ATO was more efficient in inducing a toxic response per unit of arsenic in HT-3 cells, which demonstrates the selective nature of liposomal encapsulated ATO for HPV-positive HeLa cells. This finding further supports the suitability of the optimised formulation of liposomes as the desired nanocarrier for introducing arsenic to HPV-positive cell lines. A significantly higher uptake of free ATO by HT-3 cells, compared to HeLa cells, was observed after 6 and 24 h of drug exposure. However, the arsenic concentration in HT-3 cells was then observed to have fallen after 48 h of incubation. This may be attributed to the activation of cellular efflux mechanisms in HT-3 cells. This trend was not observed in HeLa cells, with ATO concentrations continuing to increase at 48 h. It could be postulated that this was related to the presence of HPV within the cell. The mechanism behind this warrants further investigation. 

One of the main aims of employing nanotechnology in pharmaceutics is to deliver the drug specifically to the cancer cells while sparing the normal cells. There has been a lack of literature reporting on studies involving non-cancerous cell lines while investigating liposomal ATO efficacy in vitro. Hence, after establishing the effectiveness of the liposomal construct for HPV-positive cervical cancer cells, we decided to investigate the toxicity and uptake response profile of non-cancerous control cells to liposomal treatment. Two control cell lines were chosen for this study, namely human keratinocytes (HK) and human colon (CRL-1790) cells. The uptake of liposomal arsenic by HK cells was found to be higher than in the other cell lines (Figure 6a). The second control cell line, CRL-1790, displayed lower intracellular arsenic concentrations than HeLa cells after incubation with liposomal ATO (Figure 6a). However, after 24 h of treatment with liposomal encapsulated ATO, results showed that the non-cancerous cell lines were the least affected by the treatment, with cell survival rates close to 95% at the same length of incubation and the same dose of the treatment (Figure 6b). This is independent of the amount of ATO uptake by the cells. This finding suggests that liposomal-delivered ATO could be a promising candidate for targeting cervical cancer cells, because it demonstrated less toxicity to the non-cancerous cells—even at a much higher concentration. It may be the case that when liposomal-encapsulated ATO reaches the cells, it results in bio-physical changes in the cell membrane that may in turn affect the cellular transport systems. Different types of cells may react to these changes in different ways. However, HeLa cells appear to be more vulnerable to this drug treatment, which could be relevant to HPV.

The toxicity–uptake ratio indicates the toxicity induced per unit of liposomal arsenic uptake, and is an effective means of comparing the efficacy of a treatment on different cell populations. For an ideal treatment, the toxicity–uptake ratio would be low for normal cells and high for the target cancer cell population. Our results indicated that this ratio was indeed lowest for the control cell populations and highest for HeLa cells, indicating that liposomal-encapsulated ATO is a promising treatment for HPV-positive cervical cancers.

In conclusion, our current research confirms the potential of delivering ATO into HPV-infected cervical cancer cells using a liposomal drug carrier that is optimised with respect to drug loading efficiency, stability, cellular uptake, and apoptotic response by cancer cells. This nanodelivery method exhibits a better potential efficacy in the treatment of cervical cancers than using free-form ATO treatment, in that it demonstrates a higher ratio of drug toxicity to ATO uptake in the cells. Future work is warranted for evaluating whether the levels of high-risk HPV oncogenes in cervical cancer cells are reduced following this treatment and for investigating the mechanisms behind the changes. Should this prove to be the case, liposomal-encapsulated ATO may provide a novel effective therapeutic option in managing cervical cancers and possibly other HPV-associated cancers.

## 4. Materials and Methods

### 4.1. Materials

RPMI1640, l-glutamine, penicillin-streptomycin, and foetal bovine serum (FBS) were purchased from Invitrogen Life Technologies (Paisley, UK). EpiLife^®^ Medium, with 60 µM calcium; Human Keratinocyte Growth Supplement (HKGS); and nitric acid were obtained from Fisher Scientific (Loughborough, UK). Soy phosphatidylcholine (PC) and 1,2-distearoyl-sn-glycero-3-phospho-(1′-rac-glycerol) (sodium salt) (DSPG) were purchased from Avanti Polar Lipids (Alabaster, AL, USA). Methoxypolyethyleneglycol-distearoyl-phosphatidylethanolamine (DSPE-PEG2000; with mPEG MW2000Da) was obtained from Genzyme (Suffolk, UK). Cholesterol (Chol), phosphate-buffered saline (PBS), Triton-100, ATO, nickel acetate, didodecyldimethylammonium bromide (DDAB), thiazolyl blue tetrazolium bromide powder (MTT), and dialysis tubing were purchased from Sigma (Welwyn Garden City, UK). DMEM Media—GlutaMAX™, methanol, and dichloromethane were obtained from Thermofisher (Paisley, UK). An Annexin V FITC/PI apoptosis detection kit was purchased from Abcam (Cambridge, UK). 

### 4.2. Liposome Preparation and Characterization

Liposomes of different sizes were composed of soy PC, cholesterol, and DSPE-PEG2000 at a molar ratio of 54:45:1 mol %. Liposomes were prepared as has been described elsewhere [30]. Briefly, the lipids were dissolved in 1:2 methanol:dichloromethane (*v*/*v*) at room temperature. The lipid mixtures were deposited on the side wall of the rotary glass vial by removing the solvent using nitrogen. The dried lipid films were hydrated in 730 mM nickel acetate (Ni(OAc)_2_) aqueous solution for 1 h with gentle rotation. This process led to the spontaneous formation of multi-lamellar PEGylated liposomes. The liposome suspension was subsequently subjected to 10 freeze–thaw cycles (freezing in liquid nitrogen for 3 min and thawing in a 37 °C water bath for 3 min), followed by downsizing by passing through 0.1, 0.2 and 0.4 μm Anotop filters (Whatman, Cambridge, UK) to form different-sized liposomes. Extruded liposomes were dialysed against a 10 mM sodium phosphate buffer at pH 7 to get rid of unencapsulated Ni(OAc)_2_. The nickel acetate-encapsulated liposomes were then incubated with 20 mM ATO solution at room temperature for 5 h. Following the removal of unencapsulated ATO by dialysis, the concentrations of phospholipids (P), encapsulated arsenic (As), and nickel (Ni) in the liposomes were determined by an inductively coupled plasma optical emission spectrometer (ICP-OES; Thermo-Scientific iCap 6500 ICP, Stafford, UK). The molar ratios of As/P were calculated and used to determine the loading efficiency. Liposomal stability was determined by checking the As/P ratio over a period of 4 weeks, during which liposomes were stored at 4 °C in buffers of pH 7.4. Drug leakage at different pH was studied by dialysing liposomes in buffers of pH 4, pH 7 and pH 10 and assessing their loading efficiency at a period of 1, 2, 4, 6 and 24 h by measuring the As/P ratio of the samples by ICP-OES. The mean liposome sizes and zeta potential (a measure of the charges that are carried by particles that are suspended in liquid) were determined by dynamic light scattering on a Zetasizer-Nano ZS (Malvern Instruments, Malvern, UK). Liposomes of different charges were prepared as described above—with the exception of positive liposome composition, which was PC/Chol/DSPE-PEG2000/DDAB at a molar ratio of 50/33/1/16, and negative liposome composition, which was PC/Chol/DSPE-PEG2000/DSPG at molar ratio of 50/33/1/16. The positive and negative liposomes were extruded with 0.1 µm Anotop filters. The loading efficiency, stability, drug leakage, size, and zeta potential of these liposomes were investigated as outlined above. 

### 4.3. Cell Culture

Two cervical cancer cell lines, HeLa and HT-3 (ATCC, Manassas, VA, USA), and two control cell lines, human epidermal keratinocytes HK (Fisher Scientific, Loughborough, UK) and human colon epithelial cells CRL-1790 (ATCC, Manassas, VA, USA), were employed in this study. HeLa cells (HPV-18-positive) and HT-3 (HPV-negative) were cultured in RPMI 1640 complete media, and CRL-1790 were grown in DMEM media, containing 10% foetal calf serum FCS, 100 U/mL of penicillin, and 100 mg/mL streptomycin in 75 cm^2^ flasks. HK cells were cultured in Epilife media, which was supplemented with HKGS. The cells were grown in a humidified incubator containing 5% CO_2_ and 95% air at 37 °C until they reached 90% confluence. The following experiments were then set up for further studies concerning liposomal ATO exposure at different time intervals: cellular toxicity via MTT assay, flow cytometry analysis for cell apoptosis, and quantification of cellular arsenic uptake by inductively coupled plasma mass spectrometry (ICP-MS).

### 4.4. Cellular Toxicity via MTT Assay

The cytotoxicity of various liposomal formulations of ATO was determined by the MTT assay, as described previously [31]. Briefly, the experiment was set up in a 96-well plate, where the toxicity of control empty liposomes was investigated by taking an initial starting amount containing 0.5 mM of phospholipid concentration and diluting it further in a 1:10 ratio to a further six wells. The ATO encapsulating liposomes contained 30 µM of ATO in the initial sample, which was further diluted to a 1:6 ratio down to six wells. The wells were seeded with HeLa, HT-3, CRL-1790, and HK at 0.6 million cells per mL and incubated at 37 °C in the humidified incubation chamber for 24 h, 48 h and 72 h. After each time interval, the spent media was removed carefully and 50 µL of MTT solution was added into each well. After 30 min incubation at 37 °C, with 95% O_2_ and 5% CO_2_, MTT reagent was removed carefully from each well, 100 μL propanol was added to dissolve the crystals, and it was incubated at 37 °C for at least 30 min. The absorbance of this coloured solution was quantified by measuring at a wavelength of 570 nm, using a BMG LabTech FLUOstar Omega Plate Reader (Bucks, UK).

### 4.5. Cell Apoptosis by Flow Cytometry

The cells from the four different cell lines were seeded at 5 × 10^5^/mL in six-well culture plates and grown overnight. Neutral liposomes of 100 nm in size were employed for this experiment. After 24 h of treatment (media only, liposomal ATO at a dilution having 5 µM ATO encapsulated, empty liposomes at the same dilution as liposomal ATO, 5 µM free ATO), the cells were trypsinised, washed twice with PBS, and then collected into 15 mL centrifuge tubes for further staining. The cells were re-suspended in an Annexin-V binding buffer, before incubating them with fluorescein isothiocyanate (FITC)-conjugated Annexin-V and propidium iodide (PI) according to the protocol (Abcam, Cambridge, UK). All of the samples were analysed within 1 h of PI staining using BD FACSCalibur (BD, Oxford, UK).

### 4.6. Quantitative Analysis of Cellular Uptake of Arsenic by ICP-MS

Cellular uptake was analysed for 6 h, 24 h, and 48 h by inductively coupled plasma-mass spectroscopy (ICP-MS). For each analysis, the cells from the four different cell lines were seeded into sixteen 75 cm^2^ flasks at 1 × 10^6^ cells/flask, with two flasks for each treatment as duplicates. Following a 24 h cell attachment, the cells were treated as follows: control (cells in complete media), ATO lipo (5 µM ATO encapsulating liposomes), empty lipo (control liposomes without ATO), and ATO (5 µM free ATO). After 6 h treatment, the cells were washed with PBS, trypsinised, and counted before they were collected into Falcon tubes for further analysis. The cells were lysed by adding 2 mL of nitric acid while vortexing and heating at a temperature of 60 °C for 5 min, and were topped up with 8 mL of deionised water. Arsenic concentration was analysed using ICP-MS (Thermo Fisher XSeries2, Paisley, UK) and corrected to the cell number and total volume accordingly. The same procedure was carried out for different samples after 24 and 48 h incubation. 

### 4.7. Statistical Analysis

Statistical analysis described in the experimental sections was conducted using Minitab17 (Minitab Ltd., Coventry, UK). Statistical significance was determined by a two-sample *t*-test. *p* < 0.05 was considered to be significant. For flow cytometry, statistical analysis was carried out automatically through the BD Calibur software (BD Biosciences, Oxford, UK) that was provided. The mean and coefficient of variation (CV) were calculated accordingly.
